# Competition of *Escherichia coli* DNA Polymerases I, II and III with DNA Pol IV in Stressed Cells

**DOI:** 10.1371/journal.pone.0010862

**Published:** 2010-05-27

**Authors:** P. J. Hastings, Megan N. Hersh, P. C. Thornton, Natalie C. Fonville, Andrew Slack, Ryan L. Frisch, Mellanie P. Ray, Reuben S. Harris, Suzanne M. Leal, Susan M. Rosenberg

**Affiliations:** 1 Department of Molecular and Human Genetics, Baylor College of Medicine, Houston, Texas, United States of America; 2 Interdepartmental Graduate Program in Cell and Molecular Biology, Baylor College of Medicine, Houston, Texas, United States of America; 3 Departments of Biochemistry and Molecular Biology, Molecular Virology and Microbiology, and Dan L. Duncan Cancer Center, Baylor College of Medicine, Houston, Texas, United States of America; The Scripps Research Institute, United States of America

## Abstract

*Escherichia coli* has five DNA polymerases, one of which, the low-fidelity Pol IV or DinB, is required for stress-induced mutagenesis in the well-studied Lac frameshift-reversion assay. Although normally present at ∼200 molecules per cell, Pol IV is recruited to acts of DNA double-strand-break repair, and causes mutagenesis, only when at least two cellular stress responses are activated: the SOS DNA-damage response, which upregulates DinB ∼10-fold, and the RpoS-controlled general-stress response, which upregulates Pol IV about 2-fold. DNA Pol III was also implicated but its role in mutagenesis was unclear. We sought *in vivo* evidence on the presence and interactions of multiple DNA polymerases during stress-induced mutagenesis. Using multiply mutant strains, we provide evidence of competition of DNA Pols I, II and III with Pol IV, implying that they are all present at sites of stress-induced mutagenesis. Previous data indicate that Pol V is also present. We show that the interactions of Pols I, II and III with Pol IV result neither from, first, induction of the SOS response when particular DNA polymerases are removed, nor second, from proofreading of DNA Pol IV errors by the editing functions of Pol I or Pol III. Third, we provide evidence that Pol III itself does not assist with but rather inhibits Pol IV-dependent mutagenesis. The data support the remaining hypothesis that during the acts of DNA double-strand-break (DSB) repair, shown previously to underlie stress-induced mutagenesis in the Lac system, there is competition of DNA polymerases I, II and III with DNA Pol IV for action at the primer terminus. Up-regulation of Pol IV, and possibly other stress-response-controlled factor(s), tilt the competition in favor of error-prone Pol IV at the expense of more accurate polymerases, thus producing stress-induced mutations. This mutagenesis assay reveals the DNA polymerases operating in DSB repair during stress and also provides a sensitive indicator for DNA polymerase competition and choice *in vivo*.

## Introduction

There are five DNA polymerases in *Escherichia coli* (reviewed [1]). The main replicative polymerase is Pol III. The catalytic subunit, designated Pol III*, is encoded by *dnaE*. Pol I, encoded by *polA*, plays roles in processing Okazaki fragments and also in gap-filling during excision-repair processes. The other three DNA polymerases are induced to higher levels of expression by the SOS DNA-damage response [2], [3], [4]. Two of them, Pol IV and Pol V (encoded by *dinB* and *umuDC* respectively) are Y-family DNA polymerases [5]. These low-processivity error-prone polymerases play major roles in bypassing otherwise replication-blocking template lesions *via* trans-lesion synthesis and also replicate undamaged DNA [6]. Y-family polymerases have large active sites, allowing higher frequencies of base misincorporation, and no proofreading-exonuclease subunits or domains to correct insertions of incorrect bases, both leading to their lower fidelity than “housekeeping” DNA polymerases [6]. When over-expressed, Pol IV gives a mutator phenotype, causing mutations predominantly in the lagging strand [3], [7], [8]. Pol V is present at significant levels only in SOS-induced cells, and its over-expression results in slowing of DNA synthesis [9].

Pol II is encoded by *polB*. It is an accurate DNA polymerase because, like Pol I, it has an editing or proofreading 3′-exonuclease domain that can erode a mispaired primer end and so remove incorrectly inserted bases. The role of Pol II is not well defined. It has been shown to play roles in DNA replication predominantly in the lagging strand, where it might edit errors made by Pol III [10], [11]. It participates in some repair processes [12] (and reviewed by [11]) and in replication restart after DNA damage [13].

Pol III has its editing function provided by the *dnaQ-*encoded epsilon subunit of the Pol III holoenzyme. DNA polymerases are loaded onto DNA by a sliding clamp, the β-clamp, a homodimer that encircles the DNA molecule and serves as a processivity factor for DNA polymerases (reviewed by [14]). The β-clamp is the structural homologue of eukaryotic PCNA, which plays a similar role in managing DNA polymerase traffic.

It is of considerable interest to know the mechanism by which the active polymerase is chosen at any time or place during DNA synthesis. The β-clamp plays a major role in this decision, as shown by the isolation of a strain carrying a mutation in the *dnaN* gene, encoding the β-clamp, that exhibits altered preferences for DNA polymerases [15], [16]. All five DNA polymerases bind to the β-clamp, and more than one may be present on the clamp at the same time [17]. This has given rise to the concept of a toolbelt, namely that more than one polymerase is attached to the β-clamp but with only one working at a time [17], [18], [19]. Because the β-clamp is a dimer, there are two polymerase binding pockets present on a single clamp. However, there is evidence that only one polymerase is bound to a pocket at one time, and that this is the active polymerase [20]. DNA polymerases also bind to the β-clamp at sites on the clamp rim, and it is the interplay between attachment at these two sites that is believed to control polymerase choice [18], [20].

The availability of DNA polymerases also has a dramatic effect on DNA-polymerase choice, as shown by experiments in which over-expression of Pol IV stops DNA replication by displacing Pol III* [21], [22]. This is suggested to represent a Pol IV- (and SOS-) mediated checkpoint [21], as was proposed earlier for Pol V [23]. Similarly, the proofreading activity of Pol III prevents lesion bypass by translesion DNA polymerases if the translesion polymerase is not sufficiently processive [12]. This is explained as being a reaction of the proofreading activity to the persistent presence of a lesion, which will cause Pol III to remove the strand opposite the lesion whenever Pol III* attempts to extend from a primer end that is too close to the lesion [24].

The interplay of high-fidelity and low-fidelity DNA polymerases is expected to have a profound impact on mutation rates and spectra, and conversely changes in mutagenesis can provide clues about the nature of the competition between DNA polymerases. The ability of a cell to modulate the activity of low-fidelity translesion polymerases is critical to the maintenance of genomic integrity [25], but also functions to increase the mutability of stressed cells [26]. Spontaneous generation-dependent base-substitution mutagenesis has been studied in strains deficient in different polymerases in cells that lack mismatch repair so that mismatch repair does not change the spectrum of mutations observed [27]. The surprising finding was that all DNA polymerases were involved at different sites and for different basepair substitutions. For example, Pols I, III, IV or V were required for base substitutions at specific hotspots.

In this study, we looked at the roles of different DNA polymerases in stress-induced frameshift mutagenesis in the *E. coli* Lac assay [28]. Mutagenesis in this assay occurs in starving cells by −1 basepair (bp) deletions that compensate for a +1 bp frameshift mutation in a *lacIZ* fusion gene. Unlike normal DNA replication and spontaneous mutagenesis in rapidly growing cells, this mutagenesis strongly requires RpoS, the starvation- and general-stress-response regulator, and is stress-induced [29], [30]. The mutagenesis also requires single-strand DNA nicking or double-strand breakage of DNA in the general vicinity of the *lac* genes [31], the proteins of double-strand-break (DSB) repair [32], [33], [34] and DNA polymerase IV [35], [36]. Stress-induced Lac reversion also requires the SOS response [37], which is required only for producing SOS-induced levels of Pol IV [38]. The mutagenesis in this assay is an important model for mutagenesis that produces antibiotic resistant *E. coli* [39], [40], bile-resistant Salmonella [41] and several other stress-induced mutagenesis processes [26]. The working model for this mechanism of mutagenesis is that Pol IV errors occur during replication restart at sites of homologous recombinational DSB repair [26], [31]. See [26], [42] for discussion of alternatives.

In this assay, in which most frameshift reversion is clearly Pol IV-dependent, we have studied the effects of altering potential DNA polymerase competition among non-replicative as well as replicative DNA polymerases. Previous work showed that Pol V is not needed for mutagenesis in this assay [28], [37] in which frameshift reversion is measured, though it is required in a related assay for DSB-repair-protein, RpoS- and DinB-dependent mutagenesis in which base substitutions dominate [40], probably reflecting the error profiles of Pols IV and V. For Lac frameshift reversion, Pol I- [43] and Pol II- [44] defective strains both show increased mutagenesis, suggesting that these DNA Pols might compete with low-fidelity Pol IV. The involvement of Pol III* is unknown except that an antimutator allele, *dnaE915*, reduces stress-induced mutagenesis [35], [36], [44], [45], which could be caused by competition of the high-fidelity DnaE915-Pol III protein with low-fidelity Pol IV. Alternatively, it could be that (1) DNA Pol III is required for making Pol IV-dependent mutations; (2) the DnaE915-Pol III corrects (proofreads) Pol IV-generated errors; or (3) the DnaE915-Pol III* mutant protein diminishes SOS induction relative to wild-type cells, as reported [46].

We wished to determine the role of Pol III in stress-induced mutagenesis. We also wished to distinguish whether it and the other DNA polymerases compete directly with DNA Pol IV during stress-induced *lac* frameshift reversion, or cause their mutant phenotypes *via* other indirect means. At least two other explanations are possible. First, mutations affecting other DNA polymerases might alter induction of the SOS DNA-damage response, and so indirectly affect expression of *dinB*, which is required at SOS-induced levels for mutagenesis [38], thereby affecting Pol IV-dependent mutagenesis. Second, another polymerase might proofread and correct replication errors made by Pol IV. Here, we provide genetic evidence against these indirect models, and support a model in which DNA polymerases I, II, and III compete with Pol IV at sites of DNA synthesis during stress-induced mutagenesis.

## Results and Discussion

### Pol II Interferes with Pol IV-Dependent Mutagenesis by an SOS-Independent Mechanism

**Figure 1:**
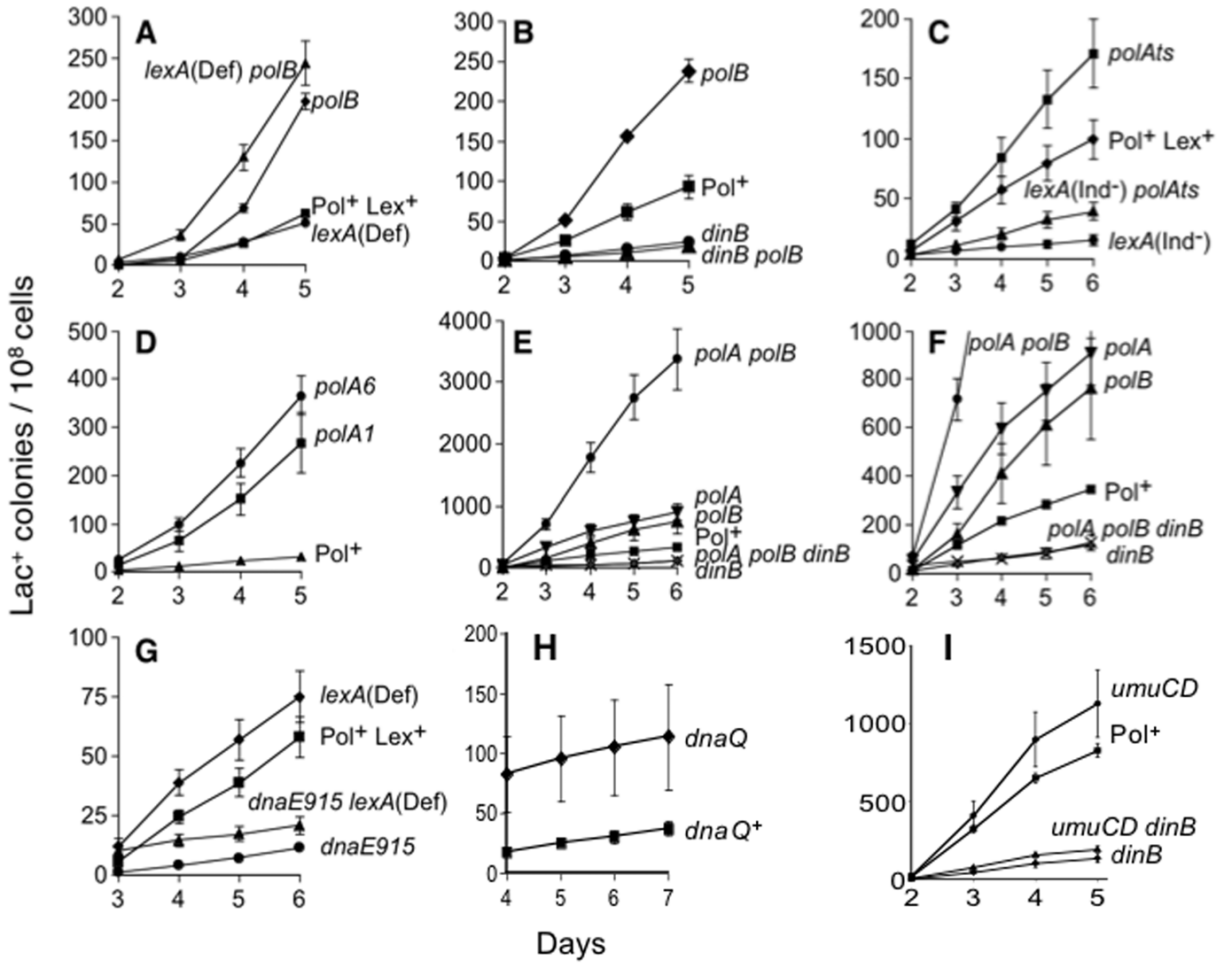
Representative examples of stress-induced mutagenesis data. See Table 1 for quantification of mutation rates from multiple experiments. (A) Loss of Pol II increases mutagenesis both in Lex^+^ and *lexA*(Def) (SOS-constitutive) cells. (B) The hypermutagenesis observed in the Δ*polB* strain is completely *dinB-*dependent. (C) Deficiency of Pol I increases Pol IV-dependent mutagenesis both in Lex^+^ and *lexA*(Ind^−^) (SOS-uninducible) cells. (D) Loss of the polymerase domain of Pol I in the *polA6* mutant increases mutagenesis. (E) Loss of Pol I and Pol II increases Pol IV-dependent mutagenesis more than the absence of either Pol I or Pol II alone. (F) Data from (E), but with the *y*-axis expanded. (G) The *dnaE915* gene product decreases mutagenesis both in Lex^+^ and *lexA*(Def) (SOS-constitutive) cells. (H) Proofreading-defective Pol III (Δ*dnaQ*) does not increase stress-induced mutagenesis. Therefore, DNA Pol III neither makes stress-induced mutations nor proofreads Pol IV errors. (I) Deletion of *umuDC* does not change stress-induced frameshift reversion in wild-type or *dinB10* cells.

**Figure 2:**
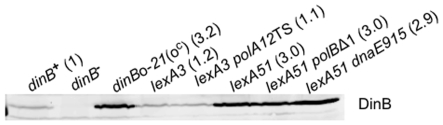
Pol IV protein levels are not increased SOS-independently in cells carrying the Δ*polB*, *polA*TS or *dnaE915* mutations. Numbers represent densitometer readings of band intensity normalized to *dinB^+^*. Strains, from left to right: SMR4562, SMR5889, SMR10308, SMR868, PH306, SMR5400, SMR8913, SMR7767. Two separate experiments gave similar results.

Strains deficient for DNA Polymerase II (Pol II, encoded by *polB*) display an increase in stress-induced Lac^+^ mutagenesis compared with Pol^+^ strains [44], [47], [48]. Therefore, the presence of Pol II inhibits stress-induced frameshift reversion. Pol II could inhibit mutagenesis by competition with the error-prone Pol IV at the site of DNA synthesis on an undamaged template. In a Pol II^-^ strain, Pol IV might gain better access to the DNA, resulting in a higher frequency of Pol IV-generated frameshift mutations than in wild-type cells. Alternatively, cells lacking Pol II might be sufficiently impaired that they induce a LexA-regulated SOS response, resulting in up-regulation of Pol IV expression, thereby indirectly increasing Pol IV-mediated mutagenesis. We tested this hypothesis using a strain with constitutive SOS induction due to a defective (null) *lexA* allele, *lexA51*(Def), and found that the *polB*Δ*1 lexA51*(Def) strain retains a hypermutagenesis phenotype equivalent to the *polB*Δ*1*strain (Figure 1A). The difference in mutation rate (per day) between *polB*Δ*1 lexA51*(Def) and its isogenic PolB^+^ control is 6.3±1.1-fold. The same fold increase is observed for *polB*Δ*1* compared with PolB^+^ in the Lex^+^ background (5.6±0.5-fold) (Table 1, detailed in Table S1). That is, the absence of Pol II still increases mutagenesis even when there is no LexA/SOS repressor and the SOS genes are maximally induced. Therefore, the effect of *polB*-defect does not promote mutagenesis by indirectly promoting cleavage of LexA and induction of the SOS genes. Whereas in principle, it might have been possible that even in the *lexA51*(Def) background, the absence of Pol II might somehow cause Pol IV levels to increase by an as-yet-unknown, SOS-independent mechanism, Western analyses show that this is not the case (Figure 2, compare *lexA*Def *polB*Δ*1* with *lexA*Def). Rather, these data support a direct competition model in which the absence of Pol II permits greater access of Pol IV to the template for DNA synthesis, promoting frameshift mutagenesis.

**Table 1:** Quantification of stress-induced mutation rates from multiple experiments.

Relevant genotypes compared^a^		Experiment^b^ identification #	Mean difference in mutation rate from control ± S.E.M.^c^	*P*-value^d^
Mutant	Isogenic control			
*polA12*(TS)	Pol^+^	1–4	2.8±0.3	0.030
*polA1*	Pol^+^	17–19	5.9±0.9	0.050
*polA6*	Pol^+^	17–19	7.3±2.1	0.050
*polB*Δ*1*	Pol^+^	5–10	5.6±0.5	0.004
*polA1 polB*Δ*1*	*polA1*	20–22	3.7±1.3	0.050
*polA1 polB*Δ*1*	*polB*Δ*1*	20–22	5.7±1.5	0.050
*polA1 polB*Δ*1dinB10*	*polA1 polB*Δ*1*	20–22	0.016±0.005	0.050
*polB*Δ*1dinB10*	*dinB10*	22–24	0.74±0.18	0.663
*dinB10*	Pol^+^	20–22	0.23±0.06	0.050
*dnaE915*	Pol^+^	11–13	0.20±0.02	0.050
*dnaQ*	DnaQ^+^	14–16	0.92±0.4	0.663
*umuDC*	Pol^+^	23–25	1.2±0.17	0.513
*umuDC dinB10*	*dinB10*	23–25	1.1±0.24	0.663
*lexA*(Ind^−^)	Lex^+^	1–4	0.29±0.09	0.043
*lexA*(Def)	Lex^+^	5–13	1.2±0.2	0.508
*polA12*(TS) *lexA*(Ind^−^)	*lexA*(Ind^−^)	1–4	3.1±0.6	0.060
*polB*Δ*1 lexA*(Def)	*lexA*(Def)	5–10	6.3±1.1	0.004
*dnaE915 lexA*(Def)	*lexA*(Def)	11–13	0.28±0.1	0.050
Other comparisons				
*polA1 polB*Δ*1*	Pol^+^	20–22	16±5	0.050
*polA1 polB*Δ*1 dinB10*	*dinB10*	20–22	0.99±0.05	1.000
*polA1*	*polA6*	17–19	1.2±0.2	0.513

**^a^See Table 2 for full strain genotypes and information. “*lexA*(Def)” strains also carry *sulA* and *psiB* mutations [49].**

**^b^Each experiment is one such as those shown in Figure 1A–I. Experiments are identified according to strains tested in parallel. Raw data for this table are given in Table S1.**

**^c^Mutation rates (mutants per day) are calculated as the number of new Lac^+^ colonies arising between days 4 and 5 (per [30]). The values listed represent the fold difference between two strains, averaged from multiple experiments in which the strains were tested in parallel, as in [49].**

**^d^*P*-values were obtained using the non-parametric Mann-Whitney Rank Sum test (SYSTAT 11 statistics software by SYSTAT Software Inc.) on rates from multiple experiments of strains tested in parallel, as in [49].**

In support of this conclusion, we found that the increased stress-induced mutagenesis observed in the Δ*polB* strain remains Pol IV-dependent (Figure 1B; also see [36]). The *dinB10* allele, encoding catalytically inactive Pol IV, reduced mutagenesis in *polB*Δ*1* cells to the level of the *dinB10* strain (Figure 1B; Table 2). These data support the hypothesis that Pol II competes with Pol IV during stress-induced mutagenesis.

**Table 2:** *Escherichia coli* K-12 strains used in this study.

Strain(s)	Relevant genotype	Reference or source
CM5407	*polA6* Tn*10*	[52]
FC29	Δ(*lac-proAB*)_XIII_ *ara thi* [F' Δ*lacIZ proAB^+^*]	[28]
FC36	Δ(*lac-proAB*)_XIII_ *ara thi* Rif^R^	[28]
FC40	FC36 [F' *lacI33*Ω*lacZ proAB^+^*]	[28]
GW2100	*umuC122*::Tn*5*	[66] CGSC^a^
MG1655	wild-type	[67]
NR9779	*dnaE486 zae*::Tn*10d*Cam	[68]
NR9915	*dnaE915 zae-502*::Tn*10*	[53]
PJH305	FC40 *fadAB3165*::Tn10Kan *lexA3*(Ind^−^)	SMR3490 x P1(SMR868)
PJH306	FC40 *polA12*(TS) *fadAB3165*::Tn*10*Kan *lexA3*(Ind^−^)	SMR3491 x P1(SMR868)
PJH354	SMR4562 *polA1 zih35*::Tn*10*	independent construct of PJH399
PJH373	SMR4562 *dinB10 polA1 zih35*::Tn*10* [F'*dinB10*]	[51]
PJH399	SMR4562 *polA1 zih35*::Tn*10*	[51]
PJH491	SMR4562 *polA1 zih35*::Tn*10 polB*Δ*1*::ΩSm-Sp	PJH354 x P1(SMR3661)
PJH510	SMR4562 *dinB10 polA1 zih35*::Tn*10 polB*Δ*1*::ΩSm-Sp [F'*dinB10*]	PJH373 x P1(SMR3661)
PJH601	FC40 Δ*umuDC*::*cat dinB10* [F'*dinB10*]	SMR5830 x P1 SMR3525
RM3980	MG1655 Δ*dnaQ903*::*tet spq-2*	[69]
RW120	Δ*umuDC*::*cat*	Roger Woodgate
SH2101	*polB*Δ*1*::ΩSm-Sp	[47]
SMR540	FC40 *dnaE486 zae*::Tn*10d*Cam	FC40 x P1(NR9779)
SMR868	FC40 *lexA3*(Ind^−^)	[37]
SMR1547	FC40 Δ*dnaQ903*::*tet spq-2 zae*::Tn*10d*Cam	FC40 x P1(SMR3640)
SMR3490	FC40 *fadAB3165*::Tn*10*Kan	[43]
SMR3491	FC40 *polA12*(TS) *fadAB3165*::Tn*10*Kan	[43]
SMR3525	FC40 Δ*umuDC*::*cat*	FC40 x RW120
SMR3640	MG1655 Δ*dnaQ903*::*tet spq-2 zae*::Tn*10d*Cam	RM3980 x P1(SMR540)
SMR3661	FC40 *polB*Δ*1*::ΩSm-Sp	FC40 x P1(SH2101)
SMR4562	independent construction of FC40	[37]
SMR5400	SMR4562 *lexA51*(Def) *sulA211 psiB*::*cat*	[37]
SMR5830	SMR4562 *dinB10* [F'*dinB10*]	[35]
SMR5889	SMR4562 Δ*dinB50*::FRT [F' Δ*dinB50*::FRT]	[35]
SMR6263	MG1655 *leu*::Tn*10*	MG1655 x P1(ZK2146)
SMR7518	SMR4562 *umuC122*::Tn*5*	SMR4562 x P1(GW2100)
SMR7767	SMR5400 *dnaE915 zae-502*::Tn*10*	SMR5400 x P1(NR9915)
SMR7768	SMR5400 *zae-502*::Tn*10*	SMR5400 x P1(NR9915)
SMR8363	SMR4562 *zae-502*::Tn*10*	SMR4562 x P1(NR9915)
SMR8365	SMR4562 *dnaE915 zae-502*::Tn*10*	SMR4562 x P1(NR9915)
SMR8913	SMR5400 *polB*Δ*1*::ΩSm-Sp	SMR5400 x P1(SMR3661)
SMR8949	SMR4562 *dinB10 polB*Δ*1*::ΩSm-Sp [F'*dinB10*]	SMR5830 x P1(SMR3661)
SMR8950	SMR4562 *dinB10 leu*::Tn*10 ara^+^* [F'*dinB10*]	SMR8949 x P1(SMR6263)
SMR8951	SMR4562*dinB10* [F'*dinB10*]	SMR8950 x P1(4562)
SMR9023	SMR4562 *polA6* Tn*10*	SMR4562 x P1(CM5407)
SMR9024	SMR4562 *polA^+^* Tn*10*	SMR4562 x P1(CM5407)
SMR10308	SMR4562 [F' *lafU2*::FRT*cat*FRT *dinBo21*(o^c^)]	[38]
ZK2146	*leu*::Tn*10*	S.E. Finkel

**^a^CGSC, *E. coli* Genetic Stock Center, Yale University.**

We note that here, as previously, when Pol IV is produced at levels equivalent to a fully derepressed SOS response, either in cells carrying the *lexA51*(Def) mutation [37], [49] or in cells with a *dinB*-operator-constitutive mutation which produces Pol IV to levels equivalent to those in *lexA51*(Def) cells [38], we observed normal, not higher-than-wild-type, levels of stress-induced mutagenesis. As concluded previously [38], this implies that cells undergoing stress-induced mutation are either fully SOS induced or are induced to levels at which some component other than Pol IV becomes rate-limiting for mutagenesis.

### Pol I Interferes with Pol IV-Dependent Mutagenesis by an SOS-Independent Mechanism

Strains deficient for DNA Polymerase I (*polA*) display elevated levels of stress-induced point mutagenesis with respect to Pol^+^ strains ([43] and Figure 1C, in which a temperature-sensitive *polA* allele was used at semi-permissive temperature), and this enhanced mutagenesis is completely Pol IV-dependent [43]. One explanation for this effect could be that Pol I, a high-fidelity DNA polymerase, could compete with Pol IV reducing its opportunities for mutagenesis. Alternatively, cells lacking Pol I are SOS induced constitutively [50], and this might increase mutagenesis indirectly by up-regulation of LexA-controlled *dinB*, as proposed [36]. We were unable to test the comparison of a *polA* mutant to Pol^+^ in a *lexA*(Def) background (that would allow constitutive *dinB* induction) because we found that this combination is not viable in this strain background. We demonstrate that the effect of mutating Pol I is not caused by enhanced SOS induction by showing that cells unable to induce the SOS genes, due to a *lexA*(Ind^−^) (“SOS-off”) mutation [1], still show increased mutagenesis when Pol I-deficient, using the *polA*TS allele at semi-permissive temperature (Figure 1C, Table 1). A null allele of *polA* renders a cell inviable in this strain background [48]. The increase in mutation rate in the *polA*TS strain over Pol^+^ is 2.8±0.3-fold per day (Table 1). The same comparison in the *lexA*(Ind^−^) background, *lexA*(Ind*^−^*) *polA*TS *versus lexA*(Ind*^−^*), yields a similar result: 3.1±0.6-fold (Table 1). Therefore the effect of *polA*TS is independent of SOS induction. The unlikely possibility that the increased mutation caused by *polA*TS results from an as-yet-undescribed SOS-independent upregulation of Pol IV levels in *polA*TS cells is ruled by Western analyses that show similar Pol IV levels in *lexA*(Ind*^−^*) and *lexA*(Ind*^−^*) *polA*TS cells (Figure 2).

### The Polymerase Domain of Pol I Inhibits Pol IV-Dependent Mutagenesis

Pol I inhibits Pol IV-dependent mutagenesis [43], but the Pol I enzymatic function responsible for inhibiting Pol IV-dependent mutagenesis was not established. Pol I has three enzymatic functions. In addition to a DNA polymerase, the protein also contains 5′-3′ (nick-translation) and 3′-5′ (proofreading) exonuclease activities. The 5′-3′ exonuclease activity is required for stress-induced gene amplification of *lac*, with no effect on point mutagenesis [51]. Mutation of both the polymerase and 3′-5′ exonuclease segments (*polA1*) results in a hypermutagenesis phenotype ([51], Figure 1D) as observed for *polA*TS (Figure 1C and [43]). One explanation for this phenotype could be that the 3′-5′ proofreading exonuclease is normally responsible for correcting errors generated by Pol IV, and in its absence, more Pol IV errors persist as mutations. We eliminated this possibility through use of the *polA6* allele, which encodes a protein deficient in the polymerization function, while retaining exonuclease functions [52]. As shown in Figure 1D, *polA6* cells retain the hypermutagenesis phenotype, supporting a model in which the polymerase portion of the protein inhibits Pol IV-dependent mutagenesis. Both *polA6* and *polA1* cells have increased mutation rates with respect to their isogenic Pol^+^ strain (7.3±2.1-fold and 5.9±0.9-fold respectively, Table 1), and their rates do not differ from each other (Figure 1D; Table 1). We conclude that the 3′-5′ exonuclease of Pol I is not involved in correcting stress-induced errors generated by Pol IV, but that the polymerase domain inhibits Pol IV-dependent mutagenesis.

### Pol I and Pol II Act Independently to Reduce Pol IV-Mediated Mutagenesis

Pol IV-dependent mutagenesis is greatly enhanced when either Pol I or Pol II are deficient. The mutation rate per day increases approximately 5-fold when either Pol I or Pol II is deficient (Table 1 and reviewed above). We report an additional approximately 5-fold increase in the *polA1 polB*Δ*1* double mutant compared with each single mutant (Figure 1E, 1F; Table 1). This implies that both Pol I and Pol II exclude lower-fidelity Pol IV from the site of DNA synthesis, largely independently. When both are absent, Pol IV has the greatest access to the site of DNA synthesis. In support of this model, the hyper-mutagenesis observed in a *polA1 polBΔ1*strain is completely *dinB*-dependent. That is, there is no difference in mutation rate between the *polA1 polB*Δ*1 dinB10* and *dinB10* strains (Figure 1E, 1F; Table 1). This indicates that Pol IV is wholly responsible for the increased mutagenesis in the *polA1 polB*Δ*1* cells. The data show that Pol I and Pol II reduce Pol IV-dependent mutagenesis and that each does so independently of the other.

### DNA Pol III Affects Pol IV-Dependent Mutagenesis Independently of SOS and Proofreading and Does Not Make the Mutations

Although Pol IV generates most of the stress-induced frameshift-reversion (“point”) mutations [35], surprisingly, a higher-fidelity “anti-mutator” mutant form of DNA polymerase Pol III, the major replicative DNA polymerase, also reduces stress-induced point mutagenesis strongly [35], [36], [44], [45]. The *dnaE915* antimutator Pol III* protein [53] could have this effect *via* any of a few mechanisms. The possibility that *dnaE915* resulted in increased availability of mismatch repair, and so less mutagenesis, was ruled out [45]. Other possibilities are that Pol III and Pol IV could work together to cause mutagenesis (perhaps one DNA polymerase causing the deletion and the other extending from the mispaired primer terminus [35]). Alternatively, reduced spontaneous SOS induction has been demonstrated in *dnaE915* cells [46], and this might lower mutagenesis by reducing expression of *dinB* encoding Pol IV. Yet another possibility is that the DnaE915-Pol III mutant protein might allow more efficient Pol III proofreading of Pol IV errors. Finally, the DnaE915-Pol III* protein might exclude the more mutagenic Pol IV better than wild-type Pol III*.

We investigated the possibility that *dnaE915* reduces Pol IV-dependent mutagenesis by reducing SOS expression in stationary-phase, stressed cells. We examined the effect of *dnaE915* in constitutively “SOS-on” *lexA-*defective strains. Constitutive de-repression of the *lex* regulon did not alleviate the decreased mutagenesis phenotype of the *dnaE915* allele relative to the *lexA*(Def) DnaE^+^ control strain (Figure 1G, Table 1). The mutation rate in *dnaE915* is ∼5-fold lower than the isogenic DnaE^+^ control, and this effect remains in the Lex-defective background (Figure 1G, Table 1). These results indicate that the decreased mutagenesis caused by *dnaE915* is independent of documented effects [46] of *dnaE915* on SOS induction.

Similarly, the unlikely possibility the *dnaE915* mutation somehow caused lower levels of Pol IV independently of effects on SOS induction is ruled by data that show similar Pol IV levels in *lexA*(Def) *dnaE915* and *lexA*(Def) strains (Figure 2). Therefore, *dnaE915* did not reduce Pol IV-dependent mutagenesis by decreasing Pol IV levels in these cells.

DnaE915-Pol III could decrease Pol IV-dependent mutagenesis by preventing Pol IV from making errors, promoting DNA Pol III proofreading of DNA Pol IV errors, reducing Pol III ability to extend synthesis from the mismatched primer terminus caused by Pol IV errors or by reducing Pol III errors that Pol IV might extend [35]. We exclude the possibility that Pol III makes a subset of the errors that become mutations by showing that a proofreading-defective Pol III (Δ*dnaQ*) does not increase stress-induced mutagenesis (Figure 1H, Table 1). Although the absolute number of Lac^+^ colonies in Figure 1H is significantly higher in the *dnaQ* strain compared with DnaQ^+^, due to a high mutation rate during liquid growth of the cultures prior to starvation on the lactose plates, the rate of Lac^+^ colony formation post-plating (the slope of the lines in Figure 1H) is the same for *dnaQ^+^* and *dnaQ^−^* strains after day 4, after which time colonies from pre-existing mutant cells cease to arise. Thus, the stress-induced-mutation rate is no higher in the *dnaQ* strain. If Pol III generated the errors that become mutations, then proofreading-defective Pol III would produce more, which it does not. Moreover, these data also demonstrate that Pol III does not proofread errors made by Pol IV, or any other DNA polymerase generating stress-induced point mutations. It has been proposed that the exonuclease activity of Pol III might be down-regulated during SOS induction [24], which could render a Δ*dnaQ* mutation inconsequential in an assay in which SOS induction plays a role, which would be compatible with our results.

The data presented indicate that Pol III neither makes nor corrects DNA polymerase errors that become stress-induced point mutations and that the *dnaE915* product does not suppress mutagenesis *via* suppression of the SOS response and down-regulation of *dinB*. Rather, we suggest that cells carrying the *dnaE915* allele show decreased stress-induced point mutagenesis because of the DnaE915-Pol III* protein's increased ability to exclude Pol IV (relative to wild-type Pol III*) and other DNA polymerases (as suggested for Pol II [44]) from the site of DNA synthesis during DSB-repair associated stress-induced mutagenesis.

### Little Effect of UmuDC on Stress-Induced Frameshift Reversion

In previous studies, Pol V (encoded by *umuDC*) did not contribute to stress-induced reversion of the frameshift allele in the Lac assay [28], [37], which we also observe here (Figure 1I, Table 1). The slightly higher rate observed in Figure 1I is not significant averaged over 5 experiments (Table 1). We find that mutagenesis in the *umuDC* strain requires *dinB* to about the same extent as the wild-type (Figure 1I and Table 1) indicating that there was no subtle effect of the absence of Pol V, such as changing the DNA polymerase responsible for the frameshift reversions measured in this assay.

Although we found no effect of Pol V in *lac* frameshift reversion in this study (Figure 1I, Table 1, and [28], [37]), previous data indicate that Pol V is also present in the DSB-repair synthesis that leads to DSB-repair-associated stress-induced mutagenesis, but simply does not contribute to −1 bp deletions. In an assay measuring stress-induced β-lactam-resistance mutagenesis in the chromosomal *ampD* gene, as here the mutagenesis required DSB-repair proteins, Pol IV, and SOS and RpoS responses, implying a DSB-repair-associated stress-induced mutagenesis mechanism [40]. However, the mutagenesis also partially required Pol V [40]. The *ampD* β-lactam-resistance mutations were mostly base-substitutions, which are not detected in the Lac assay. Similarly, Cirz et al. found that base-substitution mutations conferring ciprofloxacin resistance required Pol II, Pol IV and Pol V as well as SOS induction and DSB-repair proteins [39]. The simplest interpretation is that all five DNA polymerases are routinely present at sites of stress-induced mutagenesis associated with DSB repair, but that Pol V contributes only to substitutions whereas Pol IV contributes to both frameshift and substitution mutagenesis.

### DNA Polymerases I, II and III Compete with Pol IV during Stress-Induced Mutagenesis

The data presented imply that *E. coli* DNA polymerases I, II and III compete with DNA Pol IV during double-strand-break (DSB)-repair-associated stress-induced mutagenesis. First, we have ruled out models in which the absence of Pols I and II, or an altered function allele of Pol III, affect mutagenesis indirectly by affecting the level of SOS-induction and thus *dinB* expression (Figure 1A,C,G, Table 1). These models had been compelling given that the mutations affecting Pol I [50] and Pol III [46] do demonstrably affect SOS induction, which is required for stress-induced mutagenesis by virtue of it upregulation of Pol IV [37], [38]. Second, we ruled out possible SOS-independent effects of the relevant mutations in Pols I, II, and III on DinB protein levels (Figure 2). Third, we excluded the possibilities that Pol I or Pol III altered Pol IV-dependent mutation rates by proofreading and correcting Pol IV-generated errors (Figure 1D,H, Table 1). Fourth, whereas it seemed likely previously that DNA Pol III might be required for the Pol IV-dependent stress-induced mutagenesis [35], [36], [44], [45], our data exclude this possibility. We found that a Pol III mutant protein that reduces DinB-dependent stress-induced mutagenesis does not do this because Pol III itself makes or facilitates the DNA polymerase errors that become the mutations (Figure 1H, Table 1), but rather Pol III appears to act by excluding Pol IV. The data support models in which the mutation rate in this assay is a direct result of (is modulated by) DNA polymerases I, II and III competing with the lower-fidelity Pol IV.

### Multiple DNA Polymerases in Double-Strand-Break Repair During Stress

Previous work indicates that the sites of mutagenesis at which the DNA polymerases compete are sites of DSB repair *via* homologous recombination (HR). The mutagenesis requires HR-DSB repair proteins [32], [33], [34], and a DSB in the same DNA molecule in which the mutations occur [31]. DSBs made in the same molecule as *lac* stimulated DSB-repair-protein-, RpoS-, SOS- and DinB-dependent mutagenesis 6000-fold, whereas DSBs made in a different molecule in the same cell increased mutagenesis only 3-fold, strongly supporting error-prone DSB-repair models for the mutagenesis. DSB repair was high-fidelity and non-mutagenic in unstressed cells, but switched to a mutagenic mode using Pol IV either when cells were stressed and expressed both the RpoS general-stress response and the SOS DNA-damage response (which is activated in essentially all acts of DSB repair [46]) or if the RpoS response was expressed artificially in unstressed cells [31]. Those data revealed an RpoS-controlled switch from high-fidelity to mutagenic DSB repair, using Pol IV, under stress. Although other models have been considered (see [26], [42]), this basic interpretation of the data above has not, to our knowledge, been called into question and we are not aware of alternative interpretations. The data presented here imply that all of the DNA polymerases are present at the sites of DSB repair synthesis during stress.

The implication that all five DNA polymerases are present in acts of DSB repair under stress does not mean that all make the errors that become stress-induced mutations. First, of the five DNA polymerases, Pol IV is by far the most robust producer of -1 deletion errors [54], [55] as would produce frameshift reversions in Lac system [56], [57]. Second, the data presented here support models in which, at least for frameshift reversion, Pols I, II, and III compete with and dampen the mutagenic effect of Pol IV. Conversely, in DSB-repair-protein-dependent base-substitution mutagenesis conferring ciprofloxacin [39] or β-lactam resistance [40], Pols II, IV and V were all required, or Pol IV was required and Pol V partially required, respectively. Thus, the job of base-substitution mutagenesis appears to be shared. It may be that some DNA polymerases make errors that others extend at the mispaired primer terminus, protecting them from proof-reading functions of more processive polymerases [39]. Others of the five DNA polymerases might dampen mutagenesis simply by being present and competing with the mutagenic DNA polymerases, and influencing the dynamics of DNA polymerase choice thereby.

### Model

**Figure 3:**
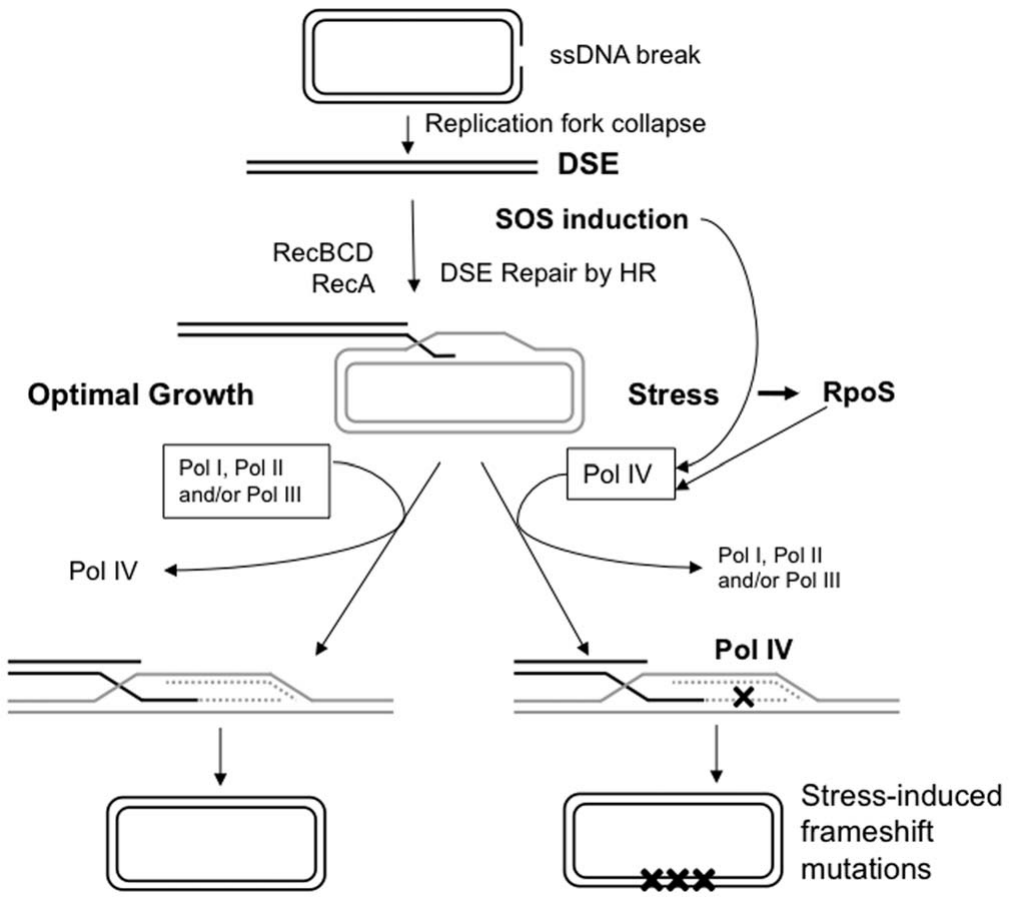
Model for the mechanism of stress-induced frameshift reversion. (Modified from [26], [31].) Double-strand ends (DSEs), formed by replication-fork collapse upon encountering a single-strand nick, are processed by RecBCD to form single-strand DNA. RecA promotes recombination with homologous DNA to initiate repair. About 40% of stationary-phase cells have two chromosomes [70], making a sister DNA molecule a probable repair partner. The 3′-invading end in the D-loop recombination intermediate primes DNA synthesis (dashed lines), and the structure is resolved by RuvABC to yield a repaired molecule. DNA synthesis can be either high- or low-fidelity, depending on the DNA polymerase(s) used: High-fidelity synthesis results from Pols I, II or III, whereas low-fidelity synthesis [yielding localized frameshift mutations (X)] results from Pol IV. Upregulation of *dinB* by SOS and RpoS stress responses results in more Pol IV molecules per cell, and possibly a more competitive Pol IV, which successfully competes with Pols I, II, and III for the sites of DNA synthesis during DSB repair, allowing Pol IV-dependent frameshift mutagenesis.

A model for the mechanism by which DNA polymerase competition could modulate HR-DSB-repair-associated stress-induced mutagenesis is illustrated in Figure 3. In this model, in growing cells, Pols I, II and III prevent Pol IV-mediated synthesis, keeping mutation rates low (left side of Figure 3). Under stress, Pol IV levels are upregulated about 10-fold by the SOS response [3], [58] and ∼2-fold by the RpoS-controlled general or stationary-phase/starvation stress response [29]. The upregulation of Pol IV is the only contribution of the SOS response to stress-induced frameshift mutagenesis [38], whereas it is unknown whether RpoS induction ushers Pol IV into acts of DSB-repair synthesis solely by increasing Pol IV levels (mass action) or by upregulating factors that might assist Pol IV. Either way, in the model, when upregulated by SOS and upregulated/assisted by RpoS induction under stress, Pol IV may then compete more successfully with Pols I, II, and III causing increased mutagenesis under stress (right side of Figure 3). An antimutator allele of Pol III (*dnaE915*) decreases Pol IV-dependent mutagenesis [35], [36], [44], [45] and Figure 1G. Our data suggest that the *dnaE915* mutant of Pol III* is better able to exclude Pol IV from the site of DNA synthesis during stress. It is likely that wild-type Pol III also competes with Pol IV effectively during DSB repair because DSB-repair synthesis in growing cells is strongly Pol III-dependent [59].

In summary, the evidence presented suggests that all five DNA polymerases are available during stress-induced mutagenesis, and that DNA polymerases I, II, and III compete with Pol IV at the primer terminus. The mechanism(s) by which various DNA polymerases win the competition under various circumstances are important biologically for determining whether cells will survive replication-blocking lesions and/or will experience mutagenesis. These mechanisms are likely to be tightly regulated by means specific to the circumstance and stress experienced.

## Materials and Methods

### Bacterial Strains and Media

*E. coli* strains (Table 2) were constructed using standard bacteriophage P1-mediated transduction techniques [60]. Relevant genotypes were confirmed by sensitivities to UV light, antibiotic resistances, and/or PCR amplification, followed by restriction digestion or DNA sequencing. Antibiotics were used in the following concentrations (µg/ml): chloramphenicol, 25; kanamycin, 30; rifampicin, 100; spectinomycin, 50; streptomycin, 50; tetracycline, 10. All M9 media [60] also contained 10 µg/ml vitamin B1 and either 0.1% glycerol or 0.1% lactose. Luria-Bertani-Herskowitz (LBH) medium was described previously [61].

### Stress-induced mutagenesis assays

The Lac^+^ assay was performed as described [34], except that in some experiments the cultures were grown at 32°C for 3 days, instead of 37°C for 2 days, before plating on M9 lactose medium. This produces much higher mutation rates as seen in Figure 1E, F and I, but does not change the relative mutability between different strains [31]. All experiments presented had less than 2-fold net population change during days 1–3 post-plating (monitored per [34]). All strains within an individual experiment were treated similarly with respect to culture temperature and length of culture time. In the Lac assay, Lac^+^ colonies result either from −1 bp deletions that compensate for the +1 bp insertion in the *lacIZ* fusion gene [56], [57], or from tandem amplification of the leaky *lac* allele to 20–50 copies [62]. Because by day 5 of an experiment, amplification accounts for only a few percent of the Lac^+^ colonies [62], we have not corrected the numbers to subtract amplification. Therefore, the data presented show total Lac^+^ colonies arising over time. Data shown represent the means ± S.E.M. for at least four independent cultures per strain. Graphs show cumulative values.

### Western analyses of Pol IV protein levels

48-hour 5 ml M9 glycerol cultures were normalized to OD_600_ of 1.0, and 1 ml of each pelleted and resuspended in 0.1 ml of Laemmli buffer [63] and boiled for 5 minutes. 15 µl from each sample were separated by discontinuous SDS-PAGE on a 14% Tris-glycine gel, and transferred to 0.2 micron Hybond LFP PVDF in 1x Dunn's modified carbonate buffer (10 mM NaHCO_3_, 3 mM Na_2_CO_3_, pH 9.9) in 20% methanol at 100 volts for 2 hr at 4°C [64]. The membrane was incubated in blocking buffer (PBS-T [137 mM NaCl, 2.7 mM KCl, 4.3 mM Na_2_HPO_4_, 1.4 mM KH_2_PO_4_ 0.1% Tween-20], 5% milk) for 1 hr, washed twice in PBS-T, treated with primary antibody [65], diluted 1∶2000 in blocking buffer, for 1 hr, washed twice in PBS-T, then treated with secondary antibody (Goat anti-rabbit IgG-Cy5, GE healthcare), diluted 1∶2000 in blocking buffer for one hour with agitation. The membrane was washed four times in PBS-T, three times in PBS (137 mM NaCl, 2.7 mM KCl, 4.3 mM Na_2_HPO_4_, 1.4 mM KH_2_PO_4_), and dried at 37°C for one hour. Cy5 fluorescence was detected on a Typhoon 9410 scanner (PMT set to 400). Western blot densitometry was performed using Adobe Photoshop.

## Supporting Information

Table S1 Details of stress-induced-mutation experiments summarized in Table 1.
(0.19 MB DOC)

## References

[1] Friedberg EC, Walker GC, Siede W, Wood RD, Schultz RA (2005). DNA Repair and Mutagenesis..

[2] Bonner CA, Hays S, McEntee K, Goodman MF (1990). DNA polymerase II is encoded by the DNA damage-inducible dinA gene of *Escherichia coli*.. Proc Natl Acad Sci U S A.

[3] Kim S-Y, Maenhaut-Michel G, Yamada M, Yamamoto Y, Matsui K (1997). Multiple pathways for SOS-induced mutagenesis in *Escherichia coli*: an SOS gene product (DinB/P) enhances frameshift mutations in the absence of any exogenous agents that damage DNA.. Proc Natl Acad Sci U S A.

[4] Sommer S, Knezevic J, Bailone A, Devoret R (1993). Induction of only one SOS operon, umuDC, is required for SOS mutagenesis in Escherichia coli.. Mol Gen Genet.

[5] Ohmori H, Friedberg EC, Fuchs RPP, Goodman MF, Hanaoka F (2001). The Y-family of DNA polymerases.. Mol Cell.

[6] Nohmi T (2006). Environmental stress and lesion-bypass DNA polymerases.. Annu Rev Microbiol.

[7] Wagner J, Nohmi T (2000). *Escherichia coli* DNA polymerase IV mutator activity: genetic requirements and mutational specificity.. J Bacteriol.

[8] Kuban W, Banach-Orlowska M, Bialoskorska M, Lipowska A, Schaaper RM (2005). Mutator phenotype resulting from DNA polymerase IV overproduction in *Escherichia coli*: preferential mutagenesis on the lagging strand.. J Bacteriol.

[9] Marsh L, Walker GC (1985). Cold sensitivity induced by overproduction of UmuDC in *Escherichia coli*.. J Bacteriol.

[10] Banach-Orlowska M, Fijalkowska IJ, Schaaper RM, Jonczyk P (2005). DNA polymerase II as a fidelity factor in chromosomal DNA synthesis in *Escherichia coli*.. Mol Microbiol.

[11] Gawel D, Pham PT, Fijalkowska IJ, Jonczyk P, Schaaper RM (2008). Role of accessory DNA polymerases in DNA replication in Escherichia coli: analysis of the dnaX36 mutator mutant.. J Bacteriol.

[12] Fujii S, Fuchs RP (2004). Defining the postion of the switches between replicative and bypass DNA polymerases.. EMBO J.

[13] Rangarajan S, Woodgate R, Goodman MF (1999). A phenotype for enigmatic DNA polymerase II: a pivotal role for pol II in replication restart in UV-irradiated *Escherichia coli*.. Proc Natl Acad Sci U S A.

[14] Johnson A, O'Donnell M (2005). Cellular DNA replicases: components and dynamics at the replication fork.. Annu Rev Biochem.

[15] Maul RW, Sutton MD (2005). Roles of the Escherichia coli RecA protein and the global SOS response in effecting DNA polymerase selection in vivo.. J Bacteriol.

[16] Sutton MD (2004). The *Escherichia coli dnaN159* mutant displays altered DNA polymerase usage and chronic SOS induction.. J Bacteriol.

[17] Indiani C, McInerney P, Georgescu R, Goodman MF, O'Donnell M (2005). A sliding-clamp toolbelt binds high- and low-fidelity DNA polymerases simultaneously.. Mol Cell.

[18] Bunting KA, RS M, Pearl LH (2003). Structural basis for recruitment of translesion DNA polymerase Pol IV/DinB to the beta-clamp.. EMBO J.

[19] Pages V, Fuchs RPP (2002). How DNA lesions are turned into mutations within cells.. Oncogene.

[20] Heltzel JM, Maul RW, Scouten Ponticelli SK, Sutton MD (2009). A model for DNA polymerase switching involving a single cleft and the rim of the sliding clamp.. Proc Natl Acad Sci U S A.

[21] Uchida K, Furukohri A, Shinozaki Y, Mori T, Ogawara D (2008). Overproduction of *Escherichia coli* DNA polymerase DinB (Pol IV) inhibits replication fork progression and is lethal.. Mol Microbiol.

[22] Furukohri A, Goodman MF, Maki H (2008). A dynamic polymerase exchange with Escherichia coli DNA polymerase IV replacing DNA polymerase III on the sliding clamp.. J Biol Chem.

[23] Opperman T, Murli S, Smith BT, Walker GC (1999). A model for a umuDC-dependent prokaryotic DNA damage checkpoint.. Proc Natl Acad Sci U S A.

[24] Pages V, Janel-Bintz R, Fuchs RP (2005). Pol III proofreading activity prevents lesion bypass as evidenced by its molecular signature within E.coli cells.. J Mol Biol.

[25] Yang W, Woodgate R (2007). What a difference a decade makes: insights into translesion DNA synthesis.. Proc Natl Acad Sci U S A.

[26] Galhardo RS, Hastings PJ, Rosenberg SM (2007). Mutation as a stress response and the regulation of evolvability.. Crit Rev Biochem Mol Biol.

[27] Curti E, McDonald JP, Mead S, Woodgate R (2009). DNA polymerase switching: effects on spontaneous mutagenesis in *Escherichia coli*.. Mol Microbiol.

[28] Cairns J, Foster PL (1991). Adaptive reversion of a frameshift mutation in *Escherichia coli*.. Genetics.

[29] Layton JC, Foster PL (2003). Error-prone DNA polymerase IV is controlled by the stress-response sigma factor, RpoS, in *Escherichia coli*.. Mol Microbiol.

[30] Lombardo M-J, Aponyi I, Rosenberg SM (2004). General stress response regulator RpoS in adaptive mutation and amplification in *Escherichia coli*.. Genetics.

[31] Ponder RG, Fonville NC, Rosenberg SM (2005). A switch from high-fidelity to error-prone DNA double-strand break repair underlies stress-induced mutation.. Mol Cell.

[32] Harris RS, Longerich S, Rosenberg SM (1994). Recombination in adaptive mutation.. Science.

[33] Foster PL, Trimarchi JM, Maurer RA (1996). Two enzymes, both of which process recombination intermediates, have opposite effects on adaptive mutation in *Escherichia coli*.. Genetics.

[34] Harris RS, Ross KJ, Rosenberg SM (1996). Opposing roles of the Holliday junction processing systems of *Escherichia coli* in recombination-dependent adaptive mutation.. Genetics.

[35] McKenzie GJ, Lee PL, Lombardo M-J, Hastings PJ, Rosenberg SM (2001). SOS mutator DNA polymerase IV functions in adaptive mutation and not adaptive amplification.. Mol Cell.

[36] Foster PL (2000). Adaptive mutation in *Escherichia coli*.. Cold Spring Harb Symp Quant Biol.

[37] McKenzie GJ, Harris RS, Lee PL, Rosenberg SM (2000). The SOS response regulates adaptive mutation.. Proc Natl Acad Sci U S A.

[38] Galhardo RS, Do R, Yamada M, Friedberg EC, Hastings PJ (2009). DinB upregulation is the sole role of the SOS response in stress-induced mutagenesis in *Escherichia coli*.. Genetics.

[39] Cirz RT, Chin JK, Andes DR, de Crecy-Lagard V, Craig WA (2005). Inhibition of mutation and combating the evolution of antibiotic resistance.. PLoS Biol.

[40] Petrosino JF, Galhardo RS, Morales LD, Rosenberg SM (2009). Stress-induced beta-lactam antibiotic resistance mutation and sequences of stationary-phase mutations in the *Escherichia coli* chromosome.. J Bacteriol.

[41] Prieto AI, Ramos-Morales F, Casadesus J (2006). Repair of DNA damage induced by bile salts in Salmonella enterica.. Genetics.

[42] Roth JR, Kugelberg E, Reams AB, Kofoid E, Andersson DI (2006). Origin of mutations under selection: the adaptive mutation controversy.. Annu Rev Microbiol.

[43] Hastings PJ, Slack A, Petrosino JF, Rosenberg SM (2004). Adaptive amplification and point mutation are independent mechanisms: Evidence for various stress-inducible mutation mechanisms.. PLoS Biol.

[44] Foster PL, Gudmundsson G, Trimarchi JM, Cai H, Goodman MF (1995). Proofreading-defective DNA polymerase II increases adaptive mutation in *Escherichia coli*.. Proc Natl Acad Sci U S A.

[45] Harris RS, Bull HJ, Rosenberg SM (1997). A direct role for DNA polymerase III in adaptive reversion of a frameshift mutation in *Escherichia coli*.. Mutat Res.

[46] Pennington JM, Rosenberg SM (2007). Spontaneous DNA breakage in single living cells of *Escherichia coli*.. Nature Genetics.

[47] Escarceller M, Hicks J, Gudmundson G, Trump C, Tonat D (1994). Involvement of *Escherichia coli* DNA polymerase II in response to oxidative damage and adaptive mutation.. J Bacteriol.

[48] Harris RS (1997). On a Molecular Mechanism of Adaptive Mutation in *Escherichia coli* [Ph.D. Thesis]..

[49] He AS, Rohatgi PR, Hersh MN, Rosenberg SM (2006). Roles of E. coli double-strand-break-repair proteins in stress-induced mutation.. DNA Repair (Amst).

[50] Bates H, Randall SK, Rayssiguier C, Bridges BA, Goodman MF (1989). Spontaneous and UV-induced mutations in *Escherichia coli* K-12 strains with altered or absent DNA polymerase I.. J Bacteriol.

[51] Slack A, Thornton PC, Magner DB, Rosenberg SM, Hastings PJ (2006). On the mechanism of gene amplification induced under stress in *Escherichia coli*.. PLoS Genetics.

[52] Joyce CM, Fujii DM, Laks HS, Hughes CM, Grindley ND (1985). Genetic mapping and DNA sequence analysis of mutations in the *polA* gene of *Escherichia coli*.. J Mol Biol.

[53] Fijalkowska IJ, Dunn RL, Schaaper RM (1993). Mutants of *Escherichia coli* with increased fidelity of DNA replication.. Genetics.

[54] Kobayashi S, Valentine MR, Pham P, O'Donnell M, Goodman MF (2002). Fidelity of Escherichia coli DNA polymerase IV. Preferential generation of small deletion mutations by dNTP-stabilized misalignment.. J Biol Chem.

[55] Tang M, Pham P, Shen X, Taylor JS, O'Donnell M (2000). Roles of *E. coli* DNA polymerases IV and V in lesion-targeted and untargeted SOS mutagenesis.. Nature.

[56] Foster PL, Trimarchi JM (1994). Adaptive reversion of a frameshift mutation in *Escherichia coli* by simple base deletions in homopolymeric runs.. Science.

[57] Rosenberg SM, Longerich S, Gee P, Harris RS (1994). Adaptive mutation by deletions in small mononucleotide repeats.. Science.

[58] Courcelle J, Khodursky A, Peter B, Brown PC, Hanawalt PC (2001). Comparative gene expression profiles following UV exposure in wild-type and SOS-deficient *Escherichia coli*.. Genetics.

[59] Motamedi M, Szigety SK, Rosenberg SM (1999). Double-strand-break repair in *Escherichia coli*: physical evidence for a DNA replication mechanism *in vivo*.. Genes Dev.

[60] Miller JH (1992). A Short Course in Bacterial Genetics..

[61] Torkelson J, Harris RS, Lombardo M-J, Nagendran J, Thulin C (1997). Genome-wide hypermutation in a subpopulation of stationary-phase cells underlies recombination-dependent adaptive mutation.. EMBO J.

[62] Hastings PJ, Bull HJ, Klump JR, Rosenberg SM (2000). Adaptive amplification: an inducible chromosomal instability mechanism.. Cell.

[63] Laemmli UK (1970). Cleavage of structural proteins during the assembly of the head of bacteriophage T4.. Nature.

[64] Dunn SD (1986). Effects of the modification of transfer buffer composition and the renaturation of proteins in gels on the recognition of proteins on Western blots by monoclonal antibodies.. Anal Biochem.

[65] Kim SR, Matsui K, Yamada M, Gruz P, Nohmi T (2001). Roles of chromosomal and episomal dinB genes encoding DNA pol IV in targeted and untargeted mutagenesis in *Escherichia coli*.. Mol Genet Genomics.

[66] Elledge SJ, Walker GC (1983). Proteins required for ultraviolet light and chemical mutagenesis. Identification of the products of the *umuC* locus of *Escherichia coli*.. J Mol Biol.

[67] Blattner FR, Plunkett G, Bloch CA, Perna NT, Burland V (1997). The complete genome sequence of *Escherichia coli* K-12.. Science.

[68] Fijalkowska IJ, Dunn RL, Schaaper RM (1997). Genetic requirements and mutational specificity of the Escherichia coli SOS mutator activity.. J Bacteriol.

[69] Slater SC, Lifsics MR, O'Donnel M, Maurer R (1994). *holE*, the gene coding for the θ subunit of Polymerase III of *Escherichia coli*: characterization of a *holE* mutant and comparison with a *dnaQ* (ε subunit) mutant.. J Bacteriol.

[70] Akerlund T, Nordstrom K, Bernander R (1995). Analysis of cell size and DNA content in exponentially growing and stationary-phase batch cultures of Escherichia coli.. J Bacteriol.

