# Supplementary material for: Competition of Escherichia coli DNA Polymerases I, II and III with DNA Pol IV in Stressed Cells
Source: PLoS One. 2010 May 27;5(5):e10862. doi: 10.1371/journal.pone.0010862 (PMC2877720; doi:10.1371/journal.pone.0010862)
Supplement: Table S1 — Details of stress-induced-mutation experiments summarized in Table 1. (0.19 MB DOC) [file pone.0010862.s001.doc]

Table S1. Details of stress-induced-mutation experiments summarized in Table 1.

| Experiment | Genotype | Strain | Day 4 cumulative Lac^+^/10^8^ cells | Day 5 cumulative Lac^+^/10^8^ cells | Difference | Mean +/- S.E.M. | Genotypes compared | Fold difference | Mean +/- S.E.M. | p |
| --- | --- | --- | --- | --- | --- | --- | --- | --- | --- | --- |
| 1 | *polA12*TS | PJH349 | 84 | 133 | 48.43 | 72.05 +/-17.41 | *polA12*TS to Pol^+^ | 2.19 | 2.78 +/-0.27 | 0.030 |
| 2 | *polA12*TS | PJH349 | 127 | 240 | 112.27 |  |  | 2.94 |  |  |
| 3 | *polA12*TS | PJH349 | 82 | 120 | 38.02 |  |  | 3.46 |  |  |
| 4 | *polA12*TS | PJH349 | 138 | 228 | 89.50 |  |  | 2.54 |  |  |
| 1 | Pol^+^ | SMR3490 | 57 | 79 | 22.12 | 26.63 +/- 6.28 |  |  |  |  |
| 2 | Pol^+^ | SMR3490 | 36 | 74 | 38.22 |  |  |  |  |  |
| 3 | Pol^+^ | SMR3490 | 26 | 37 | 10.99 |  |  |  |  |  |
| 4 | Pol^+^ | SMR3490 | 84 | 120 | 35.20 |  |  |  |  |  |
| 1 | *polA12*TS *lexA*Ind^-^ | PJH306 | 20 | 32 | 12.05 | 20.26 +/- 6.94 | polA12TS lexAInd^-^ to lexAInd^-^ | 4.44 | 3.10 +/- 0.57 | 0.06 |
| 2 | *polA12*TS *lexA*Ind^-^ | PJH306 | 26 | 44 | 17.51 |  |  | 2.72 |  |  |
| 3 | *polA12*TS *lexA*Ind^-^ | PJH306 | 18 | 28 | 10.32 |  |  | 1.76 |  |  |
| 4 | *polA12*TS *lexA*Ind^-^ | PJH306 | 63 | 103 | 40.37 |  |  | 3.48 |  |  |
| 1 | *lexA*Ind^-^ | PJH305 | 9 | 12 | 2.71 | 6.65 +/- 1.84 | *lexA*Ind^-^ to Lex^+^ | 0.12 | 0.29 +/- 0.09 | 0.043 |
| 2 | *lexA*Ind^-^ | PJH305 | 8 | 14 | 6.44 |  |  | 0.17 |  |  |
| 3 | *lexA*Ind^-^ | PJH305 | 9 | 15 | 5.85 |  |  | 0.53 |  |  |
| 4 | *lexA*Ind^-^ | PJH305 | 39 | 50 | 11.58 |  |  | 0.33 |  |  |
| 5 | *polB* | SMR3661 | 85 | 215 | 130.15 | 169.13 +/- 17.41 | *polB to* Pol^+^ | 5.41 | 5.57 +/- 0.50 | 0.004 |
| 6 | *polB* | SMR3661 | 187 | 401 | 213.66 |  |  | 5.39 |  |  |
| 7 | *polB* | SMR3661 | 400 | 623 | 223.36 |  |  | 5.42 |  |  |
| 8 | *polB* | SMR3661 | 252 | 431 | 179.89 |  |  | 6.92 |  |  |
| 9 | *polB* | SMR3661 | 74 | 212 | 138.49 |  |  | 6.75 |  |  |
| 10 | *polB* | SMR3661 | 69 | 198 | 129.20 |  |  | 3.55 |  |  |
| 5 | Pol^+^ | SMR4562 | 29 | 53 | 24.06 | 31.30 +/- 3.60 |  |  |  |  |
| 6 | Pol^+^ | SMR4562 | 43 | 83 | 39.64 |  |  |  |  |  |
| 7 | Pol^+^ | SMR4562 | 116 | 157 | 41.20 |  |  |  |  |  |
| 8 | Pol^+^ | SMR4562 | 57 | 83 | 26.01 |  |  |  |  |  |
| 9 | Pol^+^ | SMR4562 | 21 | 41 | 20.53 |  |  |  |  |  |
| 10 | Pol^+^ | SMR4562 | 27 | 63 | 36.39 |  |  |  |  |  |
| 5 | *polB*∆*1 lexA*Def | SMR8913 | 116 | 207 | 90.65 | 230.89 +/- 45.59 | *polB1 lexA*Def to *lexA*Def | 2.28 | 6.26 +/- 1.05 | 0.004 |
| 6 | *polB*∆*1 lexA*Def | SMR8913 | 415 | 637 | 222.67 |  |  | 5.28 |  |  |
| 7 | *polB*∆*1 lexA*Def | SMR8913 | 558 | 818 | 260.10 |  |  | 8.15 |  |  |
| 8 | *polB*∆*1 lexA*Def | SMR8913 | 654 | 996 | 342.07 |  |  | 8.71 |  |  |
| 9 | *polB*∆*1 lexA*Def | SMR8913 | 405 | 761 | 356.13 |  |  | 8.33 |  |  |
| 10 | *polB*∆*1 lexA*Def | SMR8913 | 130 | 244 | 113.73 |  |  | 4.82 |  |  |
| 5 | *lexA*Def | SMR5400 | 53 | 93 | 39.77 | 36.59 +/- 3.04 | *lexA*Def to Lex^+^ | 1.65 | 1.29 +/- 0.23 | 0.262 |
| 6 | *lexA*Def | SMR5400 | 79 | 122 | 42.20 |  |  | 1.06 |  |  |
| 7 | *lexA*Def | SMR5400 | 85 | 117 | 31.91 |  |  | 0.77 |  |  |
| 8 | *lexA*Def | SMR5400 | 114 | 153 | 39.29 |  |  | 1.51 |  |  |
| 9 | *lexA*Def | SMR5400 | 59 | 102 | 42.73 |  |  | 2.08 |  |  |
| 10 | *lexA*Def | SMR5400 | 28 | 51 | 23.61 |  |  | 0.65 |  |  |
| 11 | dnaE915 | SMR8365 | 6 | 10 | 3.25 | 4.62 +/- 0.85 | *dnaE915* to Pol^+^ | 0.23 | 0.20 +/- 0.02 | 0.050 |
| 12 | dnaE915 | SMR8365 | 21 | 25 | 4.44 |  |  | 0.15 |  |  |
| 13 | dnaE915 | SMR8365 | 8 | 14 | 6.18 |  |  | 0.21 |  |  |
| 11 | Pol^+^ | SMR8363 |  |  | 14.19 | 24.60 +/- 5.20 |  |  |  |  |
| 12 | Pol^+^ | SMR8363 |  |  | 29.94 |  |  |  |  |  |
| 13 | Pol^+^ | SMR8363 |  |  | 29.66 |  |  |  |  |  |
| 11 | *dnaE915 lexA*Def | SMR7767 | 90 | 92 | 2.72 | 4.63 +/- 0.86 | *dnaE915*  *lexA*Def to *lexA*Def | 0.15 | 0.28 +/- 0.13 | 0.050 |
| 12 | *dnaE915 lex* (def) | SMR7767 | 70 | 76 | 5.62 |  |  | 0.53 |  |  |
| 13 | *dnaE915 lexA*Def | SMR7767 | 45 | 50 | 4.74 |  |  | 0.15 |  |  |
| 11 | *lexA*Def | SMR7768 | 60 | 78 | 18.20 | 20.08 +/- 6.14 | *lexA*Def to Lex^+^ | 1.28 | 0.90 +/- 0.28 | 0.827 |
| 12 | *lexA*Def | SMR7768 | 41 | 51 | 10.50 |  |  | 1.35 |  |  |
| 13 | *lexA*Def | SMR7768 | 47 | 79 | 31.54 |  |  | 1.06 |  |  |
|  |  |  |  |  |  |  | *lexA*Def to Lex^+^ pooled | 1.65 | 1.16 +/- 0.18 | 0.508 |
|  |  |  |  |  |  |  |  | 1.06 |  |  |
|  |  |  |  |  |  |  |  | 0.77 |  |  |
|  |  |  |  |  |  |  |  | 1.51 |  |  |
|  |  |  |  |  |  |  |  | 2.08 |  |  |
|  |  |  |  |  |  |  |  | 0.65 |  |  |
|  |  |  |  |  |  |  |  | 1.28 |  |  |
|  |  |  |  |  |  |  |  | 0.35 |  |  |
|  |  |  |  |  |  |  |  | 1.06 |  |  |
| 14 | *dnaQ* | SMR1547 | 19 | 26 | 6.80 | 9.98 +/- 1.79 | *dnaQ* to Pol^+^ | 0.67 | 0.92 +/- 0.41 | 0.663 |
| 15 | *dnaQ* | SMR1547 | 83 | 96 | 13.00 |  |  | 1.71 |  |  |
| 16 | *dnaQ* | SMR1547 | 139 | 149 | 10.15 |  |  | 0.37 |  |  |
| 14 | Pol^+^ | SMR4562 | 8 | 18 | 10.15 | 15.03 +/- 6.20 |  |  |  |  |
| 15 | Pol^+^ | SMR4562 | 25 | 30 | 7.60 |  |  |  |  |  |
| 16 | Pol^+^ | SMR4562 | 116 | 138 | 27.35 |  |  |  |  |  |
| 17 | *polA6* | SMR9023 | 225 | 365 | 126.88 | 82.97 +/- 22.26 | *polA6* to *polA1* | 1.46 | 1.19 +/- 0.18 | 0.513 |
| 18 | *polA6* | SMR9023 | 89 | 144 | 54.64 |  |  | 0.85 |  |  |
| 19 | *polA6* | SMR9023 | 177 | 328 | 67.38 |  |  | 1.27 |  |  |
| 17 | *polA1* | PJH399 | 151 | 267 | 86.67 | 68.03 +/- 9.91 |  |  |  |  |
| 18 | *polA1* | PJH399 | 140 | 205 | 64.57 |  |  |  |  |  |
| 19 | *polA1* | PJH399 | 138 | 219 | 52.86 |  |  |  |  |  |
| 17 | Pol^+^ | SMR9024 | 24 | 32 | 11.24 | 11.58 +/- 0.66 | polA1 to Pol^+^ | 7.71 | 5.90 +/- 0.91 | 0.050 |
| 18 | Pol^+^ | SMR9024 | 21 | 34 | 12.87 |  |  | 5.02 |  |  |
| 19 | Pol^+^ | SMR9024 | 63 | 90 | 10.64 |  |  | 4.97 |  |  |
|  |  |  |  |  |  |  | *polA6* to Pol^+^ | 11.29 | 7.29 +/- 2.09 | 0.050 |
|  |  |  |  |  |  |  |  | 4.25 |  |  |
|  |  |  |  |  |  |  |  | 6.33 |  |  |
| 20 | *polA1* | PJH399 | 596 | 750 | 154.31 | 736 +/- 302.0 |  |  |  |  |
| 21 | *polA1* | PJH399 | 888 | 2054 | 1166.83 |  |  |  |  |  |
| 22 | *polA1* | PJH399 | 3288 | 4177 | 888.67 |  |  |  |  |  |
| 20 | Pol^+^ | SMR9024 | 215 | 282 | 67.51 | 128.47 +/- 35.66 | *polA1 polB* to *polA1* | 6.26 | 3.65 +/- 1.33 | 0.050 |
| 21 | Pol^+^ | SMR9024 | 387 | 514 | 126.89 |  |  | 2.73 |  |  |
| 22 | Pol^+^ | SMR9024 | 638 | 829 | 191.02 |  |  | 1.95 |  |  |
| 20 | *polB* | SMR3661 | 410 | 612 | 201.93 | 350.60 +/- 81.31 | *polA1 polB* to *polB* | 4.79 | 5.68 +/- 1.52 | 0.050 |
| 21 | *polB* | SMR3661 | 498 | 866 | 367.89 |  |  | 8.65 |  |  |
| 22 | *polB* | SMR3661 | 972 | 1454 | 481.98 |  |  | 3.60 |  |  |
| 20 | *polA1 polB* | PJH491 | 1785 | 2751 | 966.28 | 1962.12 +/- 649.8 | *polA1 polB* to Pol^+^ | 14.31 | 16.16 +/- 4.71 | 0.050 |
| 21 | *polA1 polB* | PJH491 | 8816 | 11999 | 3183.31 |  | *polA1 polB dinB10* to *dinB10* | 0.89 | 0.99 +/- 0.05 | 1.000 |
| 22 | *polA1 polB* | PJH491 | 5106 | 6843 | 1736.75 |  |  | 1.02 |  |  |
|  |  |  |  |  |  |  |  | 1.06 |  |  |
| 21 | *polA1 polB dinB10* | PJH510 | 60 | 81 | 21.17 | 25.36 +/- 5.14 | *polA1 polB dinB10* to *polA1 polB* | 0.022 | 0.016 +/- 0.005 | 0.050 |
| 22 | *polA1 polB dinB10* | PJH510 | 88 | 107 | 19.32 |  |  | 0.006 |  |  |
| 23 | *polA1 polB dinB10* | PJH510 | 74 | 109 | 35.58 |  |  | 0.020 |  |  |
| 21 | *dinB10* | SMR5830 | 62 | 86 | 23.78 | 25.41 +/- 4.24 | *dinB10* to Pol^+^ | 0.29 | 0.23 +/- 0.06 | 0.050 |
| 22 | *dinB10* | SMR5830 | 43 | 62 | 19.01 |  |  | 0.25 |  |  |
| 23 | *dinB10* | SMR5830 | 103 | 136 | 33.43 |  |  | 0.18 |  |  |
| 22 | *polB* | SMR3661 | 156 | 238 | 82.43 | 221.45 +/- 130.4 |  |  |  |  |
| 23 | *polB* | SMR3661 | 62 | 162 | 99.93 |  |  |  |  |  |
| 24 | *polB* | SMR3661 | 972 | 1454 | 489.98 |  |  |  |  |  |
| 22 | Pol^+^ | Smr4562 | 61 | 93 | 32.16 | 79.84 +/- 55.77 |  |  |  |  |
| 23 | Pol^+^ | SMR4562 | 17 | 33 | 16.38 |  |  |  |  |  |
| 24 | Pol^+^ | SMR4562 | 638 | 829 | 191.02 |  |  |  |  |  |
| 22 | *dinB10* | SMR5830 | 15 | 24 | 9.74 | 15.67 +/- 9.02 | *polB dinB10* to *dinB10* | 0.80 | 0.74 +/- 0.18 | 0.663 |
| 23 | *dinB10* | SMR5830 | 8 | 12 | 4.10 |  |  | 1.01 |  |  |
| 24 | *dinB10* | SMR5830 | 103 | 136 | 33.43 |  |  | 0.39 |  |  |
| 22 | *polB din10* | SMR8949 | 11 | 18 | 7.55 | 8.30 +/- 2.63 | *polB dinB10* to Pol^+^ | 0.23 | 0.19 +/- 0.06 | 0.050 |
| 23 | *polB din10* | SMR8949 | 4 | 9 | 4.16 |  |  | 0.25 |  |  |
| 24 | *polB din10* | SMR8949 | 18 | 31 | 13.19 |  |  | 0.07 |  |  |
| 25 | *umuDC* | SMR3525 | 898 | 1127 | 229.00 | 129.56 +/- 52.3 | *umuDC* to Pol^+^ | 1.30 | 1.17 +/- 0.17 | 0.513 |
| 26 | *umuDC* | SMR3525 | 601 | 709 | 107.93 |  |  | 0.83 |  |  |
| 27 | *umuDC* | SMR3525 | 245 | 306 | 51.75 |  |  | 1.38 |  |  |
| 25 | Pol^+^ | SMR4562 | 651 | 827 | 175.84 | 114.59 +/- 40.74 |  |  |  |  |
| 26 | Pol^+^ | SMR4562 | 528 | 659 | 130.52 |  |  |  |  |  |
| 27 | Pol^+^ | SMR4562 | 110 | 147 | 37.42 |  |  |  |  |  |
| 25 | *umuDC dinB10* | PJH601 | 159 | 195 | 36.13 | 24.52 +/- 7.86 | *umuDC dinB10* to *dinB10* | 1.08 | 1.05 +/- 0.24 | 0.663 |
| 26 | *umuDC dinB10* | PJH601 | 45 | 55 | 9.54 |  |  | 1.43 |  |  |
| 27 | *umuDC dinB10* | PJH601 | 58 | 86 | 27.90 |  |  | 0.62 |  |  |
| 25 | *dinB10* | SMR5830 | 103 | 136 | 33.43 | 28.37 | 11.36 |  |  |  |
| 26 | *dinB10* | SMR5830 | 24 | 30 | 6.65 |  |  |  |  |  |
| 27 | *dinB10* | SMR5830 | 73 | 118 | 45.02 |  |  |  |  |  |
